# TDS-YOLO: a lightweight detection model for fine-grained segmentation of tea leaf diseases

**DOI:** 10.3389/fpls.2026.1769143

**Published:** 2026-03-09

**Authors:** Qizhao Xie, Tao Wang, Weiwei Zu, Yusmadi Yah Jusoh, Liangquan Jia

**Affiliations:** 1 Department of Software Engineering and Information System, Faculty of Computer Science and Information Technology, Universiti Putra Malaysia, Serdang, Selangor, Malaysia; 2 School of Information Engineering, Huzhou University, Huzhou, China

**Keywords:** deep learning, EfficientHead, TDS-YOLO, tea leaf disease segmentation, YOLOv11

## Abstract

**Introduction:**

Timely identification and precise segmentation of tea leaf diseases are essential for intelligent agricultural management. However, balancing lightweight deployment and high-precision segmentation remains challenging under uneven illumination, background interference, and subtle early-stage lesion textures in natural environments.

**Methods:**

We propose TDS-YOLO, a lightweight segmentation model based on the YOLOv11 framework. The model introduces three innovations: (1) C3K2_EViM_CGLU for global dependency modeling, (2) EfficientHead for lightweight pixel-level representation, and (3) C2PSA_Mona to enhance multi-scale texture perception.

**Results:**

Experiments on a diverse dataset of 4,933 images show that TDS-YOLO achieves state-of-the-art performance with only 2.53M parameters. It reaches an mAP@0.5 of 90.1% for both detection and segmentation, outperforming YOLOv11-seg and other mainstream models while maintaining an inference speed of 96 FPS.

**Discussion:**

The proposed approach provides an efficient and robust solution for real-time monitoring of tea diseases, supporting precision tea plantation management and broader smart digital agriculture applications.

## 1 Introduction

Tea (Camellia sinensis) is among the most widely consumed non-alcoholic beverages worldwide and contains over 700 biologically active compounds—such as polyphenols, amino acids, and caffeine—that contribute positively to human health (Samanta, 2022). Beyond its nutritional value, tea cultivation is also a key economic sector in major producing countries, including China, India, and Sri Lanka, where it supports rural development and contributes to farmers’ livelihoods (Tiwari et al., 2023). However, tea plants are highly susceptible to numerous diseases, including tea anthracnose, algal leaf spot, and tea blight, which can severely impair photosynthesis, reduce yield, and deteriorate leaf quality; in extreme cases, outbreaks may cause substantial economic losses (Ahmed et al., 2018; Pandey et al., 2021). Given these challenges, there is a pressing need for efficient and reliable disease monitoring strategies. In particular, fine-grained, pixel-level segmentation methods capable of detecting early and subtle lesion symptoms are crucial for maintaining tea plantation health, improving production efficiency, and promoting intelligent agricultural management.

Traditional monitoring of tea diseases primarily relies on manual field inspection or expert visual identification. Such approaches are labor-intensive, inefficient, and insufficient to meet the needs of precision agriculture (Rahat et al., 2025). To overcome these limitations, advances in artificial intelligence have accelerated the adoption of machine learning and computer vision techniques in agricultural disease monitoring (Wang C. et al., 2024; Wang C. et al., 2025). Early research on tea disease detection mostly relied on conventional machine learning models combined with hand-crafted features describing color, texture, or shape. For example, Zhao et al. (Zhao et al., 2022) integrated continuous wavelet transforms with an SVM classifier to accurately distinguish visually similar tea disease symptoms, demonstrating the potential of handcrafted-feature-based classifiers. Liu et al. (Liu et al., 2022) utilized IoT-based environmental parameters to construct a multivariate linear regression model for statistical prediction of tea blister blight occurrence. Similarly, Saputro et al. (Saputro et al., 2020) extracted HSV color features from leaf images and applied a KNN classifier to identify three typical pest types. To further optimize the performance of such handcrafted-feature-based systems, researchers have introduced meta-heuristic approaches, such as the Salp Swarm Algorithm (SSA), to implement robust feature selection and improve the precision of plant disease detection (Xie et al., 2023). Nevertheless, these traditional models rely heavily on manually engineered features, which limits their generalization ability under complex natural conditions (Yang et al., 2023). Moreover, they are incapable of pixel-level segmentation of small disease spots, restricting fine-grained quantitative assessment of disease severity.

In recent years, the rapid development of deep learning has provided an end-to-end solution for the automated identification of tea pests and diseases. Unlike traditional models that rely on hand-crafted features, deep neural networks can directly learn discriminative representations from raw imagery, leading to more robust recognition performance (Shoaib et al., 2025; Wang S. et al., 2025). For instance, Lee et al. (Lee et al., 2020) employed a convolutional neural network (CNN) to construct a tea pest–disease detector capable of locating and identifying seven categories of leaf injuries in real field conditions. Datta et al. (Datta and Gupta, 2023) designed a deep multi-layer CNN to perform multi-class classification on datasets containing six tea diseases along with healthy samples. Sivaraman et al. (Sivaraman et al., 2025) adopted VGG16 with transfer learning to build a disease classifier for diverse tea leaf conditions. Furthermore, to address the challenge of data scarcity and domain discrepancy between different crops, advanced strategies such as deep transfer learning with mixed subdomain adaptation have been developed to enhance cross-species disease diagnosis (Yan et al., 2021). Wen et al. (Wen et al., 2025) further evaluated multiple lightweight CNN architectures and introduced an improved MnasNet equipped with SimAM attention for multi-type tea pest–disease classification. Although these models significantly enhance image-level recognition, most of them remain limited to classification tasks and thus fail to meet the practical needs of field applications, where accurate localization, counts, and spatial distribution of lesions are essential.

To address the combined need for localization and classification, research has progressively shifted toward object detection frameworks such as Faster R-CNN (Ren et al., 2017), SSD (Liu et al., 2016), and the YOLO family (Redmon et al., 2016). Among them, YOLO stands out due to its high efficiency, fast inference, and strong global perception, making it well-suited for real-time tea-plantation monitoring under resource constraints. Xue et al. (Xue et al., 2023) proposed an enhanced YOLO-Tea model based on YOLOv5, yielding approximately 0.3%–15% performance improvement over the original model. Lin et al. (Lin et al., 2023) developed TSBA-YOLO for tea disease detection, achieving superior small-object recognition accuracy and more stable real-time performance in complex backgrounds. Bao et al. (Bao et al., 2023) designed the DDMA-YOLO model for UAV-based remote detection of tea blight, improving AP@0.5 by ~3.8% and recall by ~6.5% over baseline YOLOv5. Ye et al. (Ye et al., 2024) combined image enhancement with an improved YOLOv8 to estimate disease severity, achieving ~95.26% mAP under challenging conditions such as low illumination or tiny lesions. Although these approaches have promoted significant advances in tea disease detection, they largely remain constrained to object-level detection—emphasizing “whether lesions exist” and bounding-box localization—without offering fine-grained solutions for precise boundary extraction, area estimation, or severity quantification.

Despite recent advancements, existing models still face several key challenges when dealing with complex tea plantation environments. First, backbone networks often struggle to capture long-range global contextual cues and selectively emphasize disease-relevant features, leading to fragmented or incomplete representations of weakly textured lesions. Second, conventional segmentation heads fail to balance lightweight design with precise boundary delineation, particularly for tiny and irregular lesion regions. Third, the interaction among multi-scale features frequently suffers from spatial and channel misalignment, which reduces robustness under uneven illumination and background interference.

Motivated by the above limitations, this study develops a systematic enhancement of the YOLOv11-seg pipeline, where each proposed component is explicitly designed to address one of the identified challenges:

1. we design a novel C3K2_EViM_CGLU module to jointly strengthen global dependency modeling and lesion-specific feature selectivity;
2. we propose an EfficientHead segmentation head that is both lightweight and detail-sensitive, optimized for boundary expression of tiny lesions in high-resolution tea imagery;
3. we introduce a C2PSA_Mona module to enhance long-range contextual association and multi-scale texture alignment via joint channel–spatial interaction.

The resulting TDS-YOLO framework demonstrates outstanding segmentation performance for tea pests and diseases, achieving significant improvements in fine lesion boundary extraction and tiny-spot detection, while maintaining real-time inference efficiency and offering a breakthrough in practical detection performance.

## 2 Materials and methods

### 2.1 Dataset

To construct a representative dataset for tea pest and disease segmentation, we adopt a dual-source data collection strategy. First, a portion of the images was captured on-site at a tea plantation located in Mozitan Town, Huoshan County, Lu’an City, Anhui Province, China (latitude 31.23°, longitude 111.33°). The data acquisition process encompassed diverse natural imaging conditions, including varying illumination, disease progression stages, changes in leaf pose, as well as complex background disturbances such as weeds, specular reflections, and water droplets. This ensures real-world variability representative of field environments.

Second, to expand the diversity of disease categories and enhance the heterogeneity of visual features, additional tea disease images were collected from public online resources. Only images with sufficient resolution, reliable disease visibility, and consistent source quality were retained following a strict filtering process.

**Figure 1:**
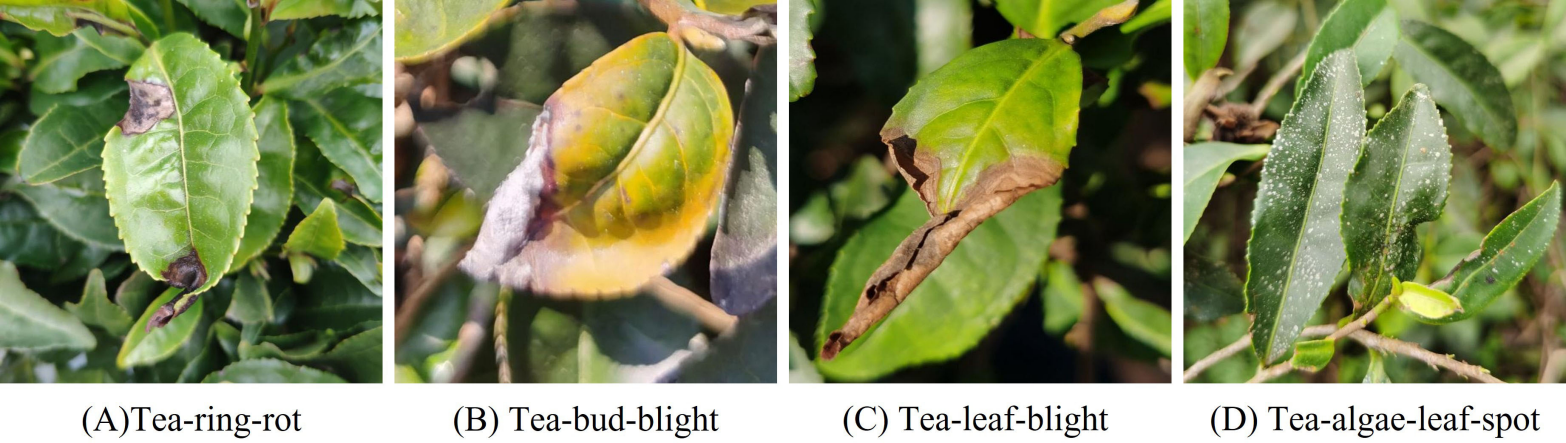
Representative samples of four tea leaf disease categories: **(a)** Tea-ring-rot, **(b)** Tea-bud-blight, **(c)** Tea-leaf-blight, and **(d)** Tea-algae-leaf-spot.

In total, the constructed dataset consists of 4,933 tea disease images spanning four common disease types. To ensure robust model training and unbiased evaluation, the dataset was randomly split into training, validation, and testing sets with a ratio of 8:1:1. The detailed distribution of samples is summarized in **Table 1**. Representative dataset samples are illustrated in **Figure 1**.

**Table 1:** Detailed distribution of the tea leaf disease dataset.

Disease category	Scientific name	Annotated name	Training	Validation	Test	Total
Tea leaf blight	Colletotrichum camelliae	tea-leaf-blight	605	75	75	755
Tea bud blight	Phyllosticta gemmiphliae	tea-bud-blight	1,151	144	144	1439
Tea ring rot	Pestalotiopsis theae	tea-ring-rot	1280	160	160	1600
Tea algae leaf spot	Cephaleuros virescens	tea-algae-leaf-spot	925	115	115	1155
Total	–	–	3961	494	478	4933

### 2.2 Overall architecture of TDS-YOLO

YOLOv11 (Khanam and Hussain, 2024) represents the latest generation of the YOLO family, whose architecture consists of four core components: the input layer, a backbone network, a feature-fusion neck, and a detection–segmentation head. The backbone adopts an enhanced CSP structure for multi-scale feature extraction, while the neck performs bi-directional (bottom-up and top-down) feature fusion to aggregate semantic information across different resolutions. The detection–segmentation head simultaneously outputs bounding boxes, class predictions, and pixel-level masks from multi-scale feature maps, thereby achieving end-to-end object detection and instance segmentation (Jegham et al., 2024; Kotthapalli et al., 2025). Compared with previous versions, YOLOv11 provides substantial improvements in inference stability, speed, and accuracy, making it a solid foundation for building a lightweight and efficient segmentation model tailored to tea-leaf disease analysis.

**Figure 2:**
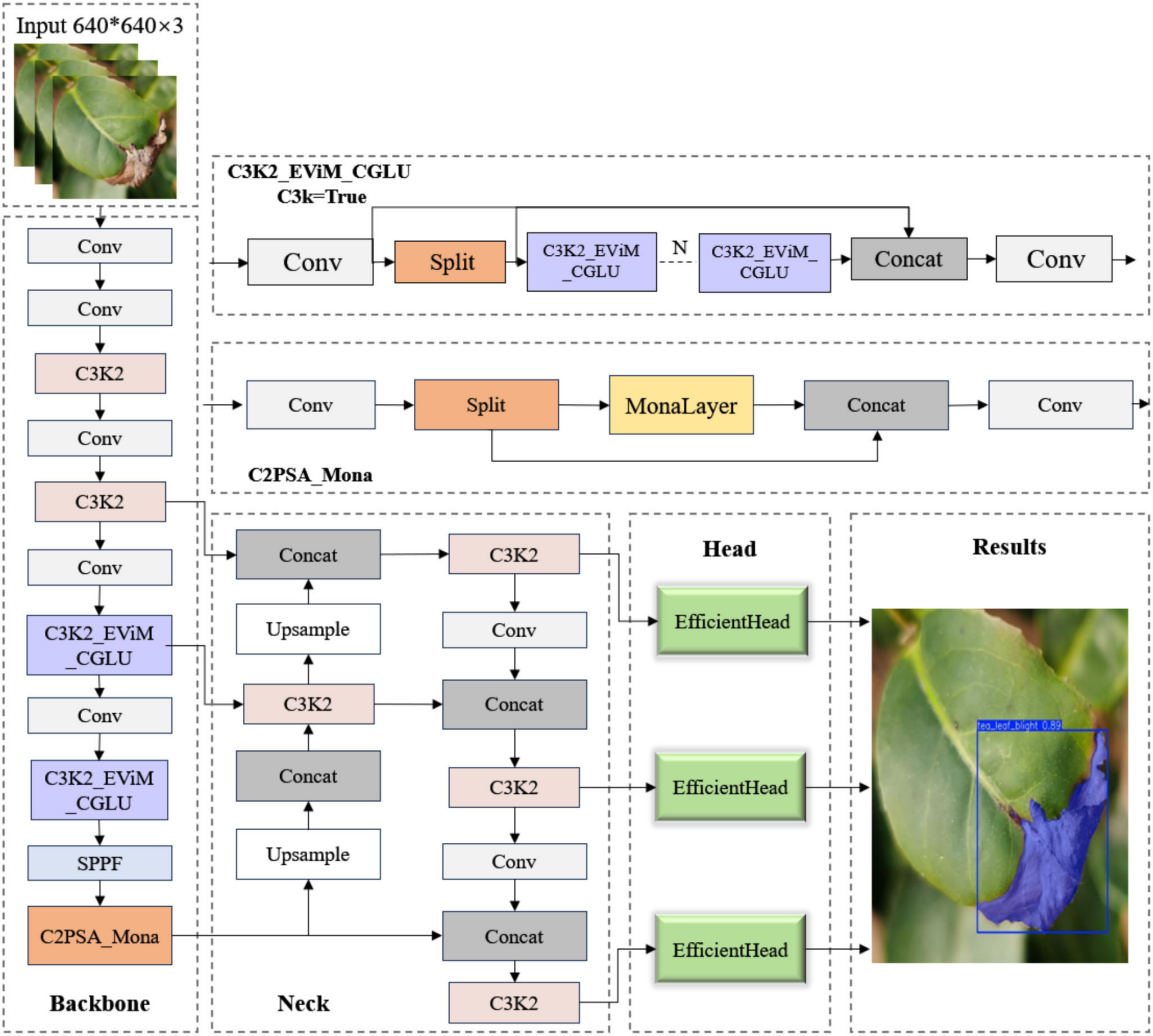
Architecture of the proposed TDS-YOLO model.

Based on the YOLOv11 framework, we propose TDS-YOLO, an optimized architecture specifically designed for fine-grained segmentation of tea pests and diseases. The model introduces targeted enhancements at three levels—backbone design, feature augmentation modules, and segmentation head construction—to improve the perception and representation of tiny lesions, weak-texture regions, and disease patterns under complex backgrounds. The overall architecture of TDS-YOLO is illustrated in **Figure 2**.

#### 2.2.1 C3K2_EViM_CGLU module

The C3k2 module is an efficient feature extraction unit built upon the Cross-Stage Partial (CSP) architecture, aiming to balance representational capacity and computational efficiency, and has been widely adopted in detection and segmentation models (Khanam and Hussain, 2024). Although the native C3k2 module in YOLOv11 performs efficient local feature extraction, its reliance on purely grouped multi-branch convolutions limits its ability to model long-range dependencies and inter-region semantic interaction. This limitation restricts its effectiveness in tea disease segmentation, where capturing fine-grained global information is essential.

From a task perspective, tea leaf disease segmentation presents two major challenges: (1) early-stage lesions are often weakly textured and spatially scattered across the leaf surface, requiring global contextual awareness beyond local receptive fields; and (2) lesion-related visual cues are highly similar to leaf veins and background noise, demanding selective feature emphasis. The original C3k2 module lacks explicit mechanisms to address these challenges. To address this issue without disrupting the original C3k2 structure, we introduce the C3K2_EViM_CGLU module as a backbone feature extractor. For clarity, C3K2_EViM_CGLU denotes a C3k2-based enhancement module that integrates EfficientViM-style global dependency modeling (EViM) with channel-gated linear units (CGLU). The proposed module integrates two key components: (1) the HSM-SSD unit from EfficientViM (Lee et al., 2025), and (2) the Convolutional GLU (ConvGLU) channel-mixing attention mechanism from TransNeXt (Shi, 2024). This design aims to significantly enhance discriminative lesion perception while maintaining computational efficiency.

In natural tea plantation environments, segmentation performance is often degraded by uneven illumination, weed interference, and reflective water droplets. To mitigate these issues, the incorporated HSM-SSD (Hidden State Mixer for State-Space Dual) replaces heavy linear projections in the pixel domain with compact latent-state interaction. This latent interaction acts as an information compressor, effectively filtering high-frequency background noise while modeling global dependencies of the entire leaf at negligible computational cost. Without such global dependency modeling, early-stage or diffuse lesions tend to be fragmented or overlooked when only local convolutional features are considered. As a result, contextual cues surrounding lesion occurrence are more accurately preserved.

**Figure 3:**
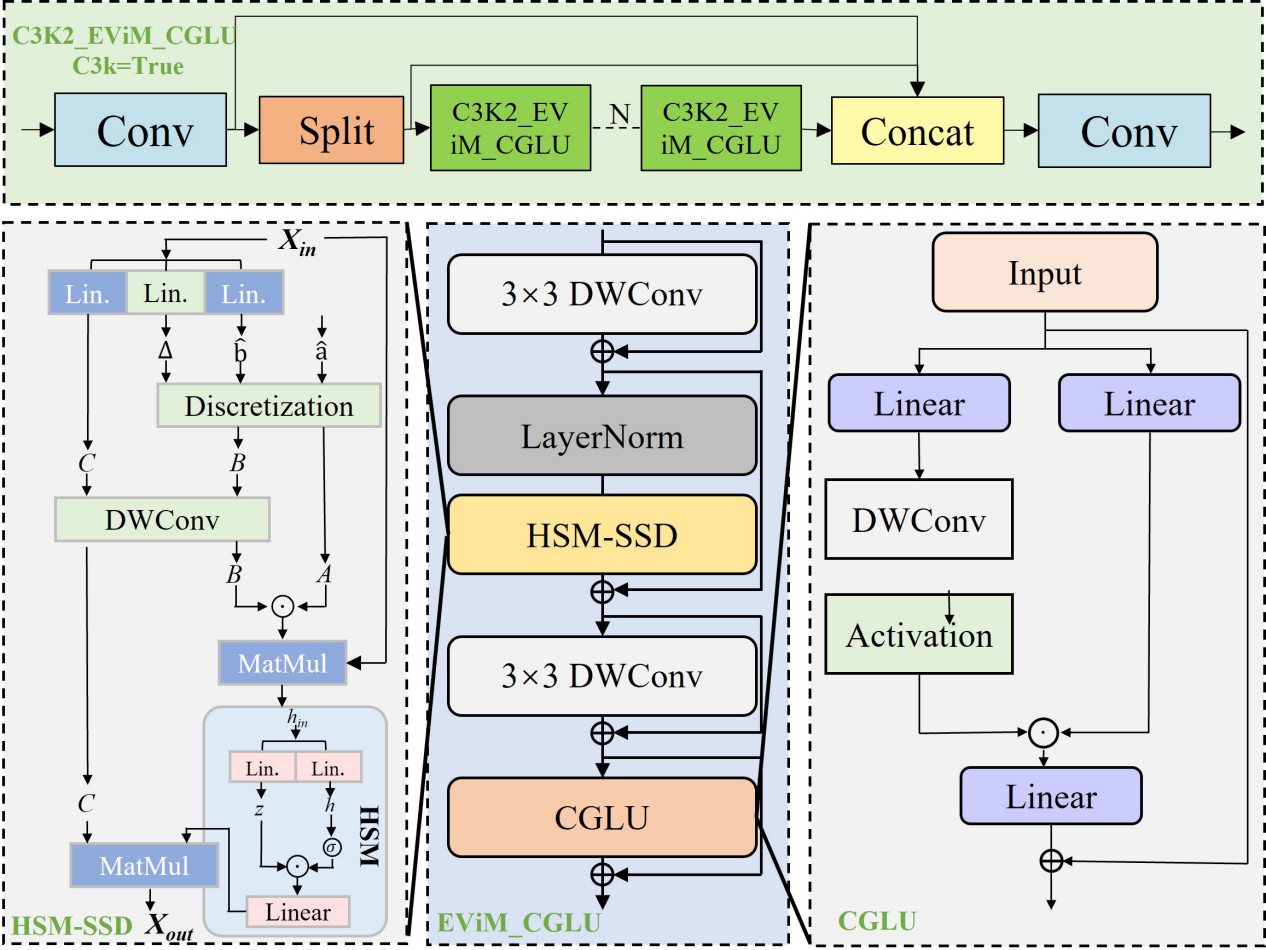
Illustration of the C3K2_EViM_CGLU module.

In early stages, tea leaf diseases typically appear as small color variations that can be easily confused with vein textures. To address this, the module employs CGLU (Channel-Gated Linear Unit), which performs parallel linear projection and gating-based channel weighting. This gating mechanism compensates for the lack of channel discrimination in global modeling by adaptively reweighting feature channels according to their relevance to disease characteristics. This enables dynamic channel selection that adaptively assigns higher weights to lesion-related color and texture channels while suppressing responses from healthy tissues. Such selective enhancement increases the contrast between diseased and healthy areas at the feature extraction stage, directly benefiting fine-grained segmentation. A schematic illustration of the C3K2_EViM_CGLU module is shown in **Figure 3**.

In the implementation, given an input feature tensor $\;F_{in}\in {\mathbb{R}}^{C\times H\times W}$, where C denotes the channel dimension and H×W represents the spatial resolution, the C3K2_EViM_CGLU module utilizes the HSM-SSD component to migrate computation from the high-dimensional pixel domain into a compact latent state space. Specifically, the input feature sequence is first aggregated into a latent state vector. To reduce computational overhead, a position-weighted linear transformation is employed instead of dense attention calculation. The state aggregation is defined as shown in Equation 1:

(1) $$S=\sum_{j=1}^{N}\omega_{j}(W_{p}\cdot f_{j})$$

where $f_{j}$ denotes the feature vector at the j-th spatial location, N is the total number of spatial positions, $W_{p}$ is the projection matrix that maps features into the latent state space, and $\omega_{j}$ is a learnable positional weight that adaptively evaluates the contribution of disease-relevant regions to global feature encoding.

To further enhance lesion-texture discrimination, the aggregated state S is modulated with a channel gating mechanism through CGLU (Channel-Gated Linear Unit) and then projected back to the original feature space to produce the output $F_{out}$. The output projection process combines nonlinear activation and element-wise gating, and is formulated as:

(2) $$F_{out}\;=\;W_{o}\cdot (\sigma (W_{g}S)⊙S)$$

where ⊙ denotes element-wise multiplication, σ(·)  is the Sigmoid activation function, and $W_{g}\;$ and $W_{o}\;$ are the gating and output projection matrices, respectively.

The essence of Equation 2 lies in its dynamic channel modulation, enabling selective suppression of features associated with healthy tissues while amplifying lesion-related responses. This gated latent-space inference significantly reduces parameter count and computation complexity without sacrificing global receptive field modeling or fine-grained lesion detail. Consequently, the proposed module markedly improves segmentation accuracy in complex natural tea plantation environments.

#### 2.2.2 Efficient and lightweight detection head

The original segmentation head of YOLOv11 is designed as an integrated detection–segmentation output module, where multi-scale decoupled detection branches are constructed for each feature level from the backbone and neck, predicting class scores and bounding-box distributions independently. In parallel, a prototype subnet operating on high-resolution feature maps generates a shared set of mask bases. The final pixel-wise segmentation masks are produced by linearly combining these prototypes with mask coefficients predicted at each scale (Khanam and Hussain, 2024). Although this architecture achieves strong accuracy in general-purpose segmentation tasks, it exhibits limitations when applied to tea pest and disease segmentation. Specifically, the scale-specific branches rely heavily on stacked standard 3×3 convolutions with dense channel connections, resulting in high parameter counts and computational complexity that hinder deployment on mobile or embedded systems frequently required in tea plantation monitoring. Moreover, the original head struggles to capture fine-grained structural cues such as tiny lesion spots or slender edge-type infections along the leaf margins. These detailed regions often produce jagged mask boundaries or are confused with natural leaf veins, which degrades the reliability of lesion area estimation and consequently affects quantitative assessment of disease severity.

**Figure 4:**
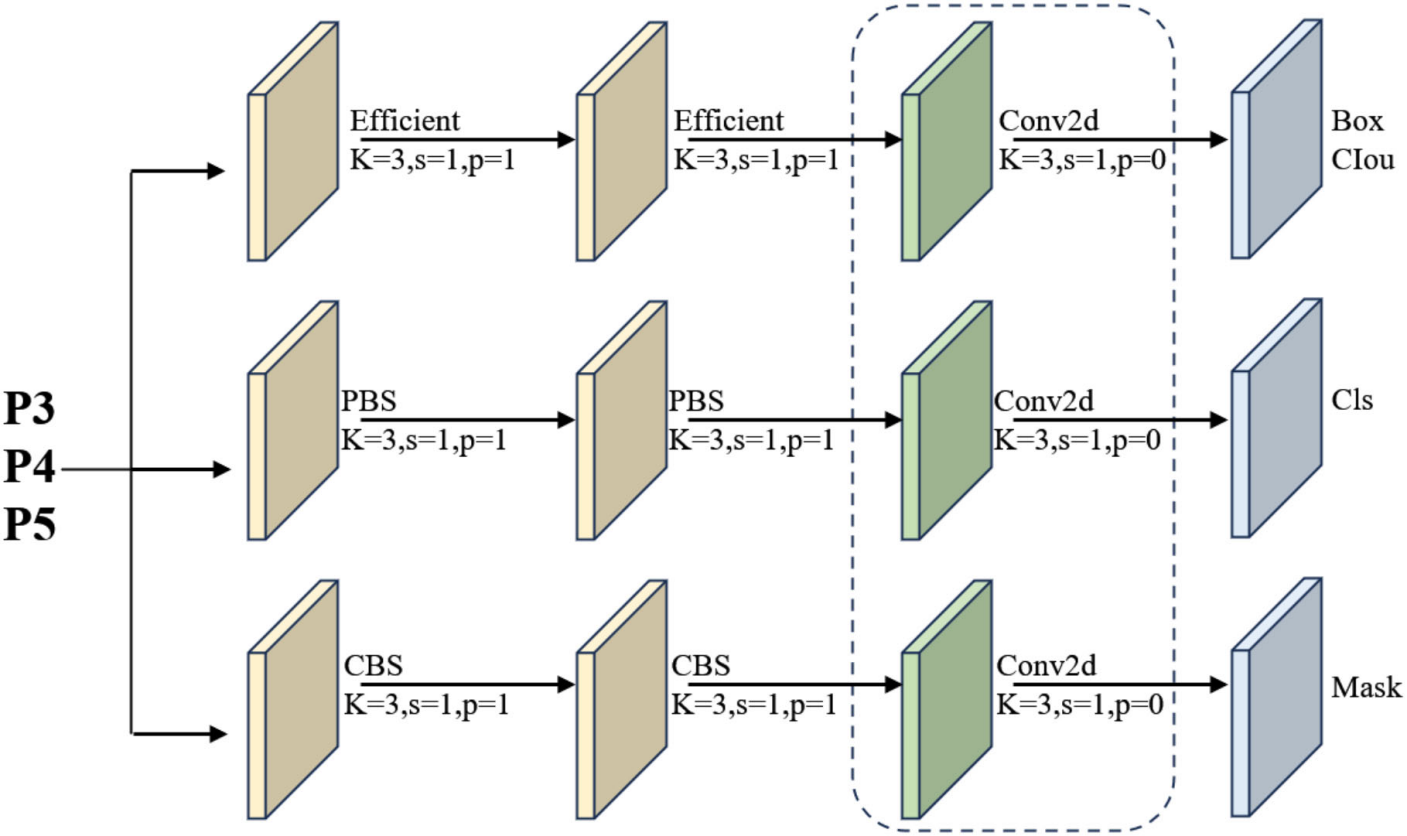
Structure of the EfficientHead module.

To remedy these limitations while retaining the unified detection–segmentation paradigm of YOLOv11, we design a lightweight and detail-sensitive segmentation head named EfficientHead, specifically tailored for tea pest and disease analysis. It should be noted that the proposed EfficientHead is not a direct reuse of an existing detection head, but a task-driven redesign of the YOLOv11 segmentation head, specifically optimized for fine-grained tea leaf disease segmentation under lightweight constraints. The proposed head first addresses redundant computation and excessive parameterization in multi-scale branches through a grouped convolution strategy embedded in the shared detection–segmentation output layer. For any scale feature map, two consecutive 3×3 grouped convolutions are employed to extract local spatial information, where each convolution only operates within 1/16 of the channel groups. This design substantially reduces channel-wise redundancy and memory access overhead without compromising the spatial receptive field. As a result, the multi-scale detection head maintains high inference throughput even with high-resolution tea leaf imagery. Each branch outputs class predictions and bounding-box distributions through 1×1 convolution, and the regression branch is further refined using Distribution Focal Loss, which aggregates discrete distributions into continuous coordinates, thereby improving boundary precision and stabilizing regression performance for small, irregular disease lesions. An overview of the EfficientHead structure is shown in **Figure 4**.

Building on this lightweight detection scaffold, we additionally integrate a prototype-based mask reconstruction mechanism to achieve fine-grained pixel segmentation of tea leaf diseases. A prototype set $P\in {\mathbb{R}}^{N_{P}\times H_{P}\times W_{P}}\;$ is generated from the highest-resolution feature map via a two-layer convolutional operator, as shown in Equation 3:

(3) $$P\;=\;f_{p}(X_{0})$$

Where $X_{0}\;$ denotes the feature map with the largest spatial resolution and $f_{p}$ (·) represents the number of prototype bases. Since disease boundaries, bite-like erosion marks, and mold spot textures often share highly consistent local geometric structures, these prototypes function as morphological bases, capturing common lesion textures and edge patterns on tea leaves.

To endow each detection scale with segmentation capability, the corresponding feature maps X predict lightweight mask coefficients, which are unfolded spatially and concatenated to form a global coefficient matrix. Instance-level masks are subsequently obtained by linearly combining the coefficients with the prototypes. This linear reconstruction not only keeps the segmentation process computationally efficient but also enables high-resolution prototypes to accurately restore lesion contours, spot shapes, and serrated bite edges. Such fidelity is crucial for differentiating corrosion-type blight, mold-like lesions, and normal green venation, particularly under complex natural tea-growing environments.

#### 2.2.3 C2PSA_Mona

Within lightweight backbone networks designed for cross-channel and cross-spatial feature interaction, the C2PSA module has been widely adopted due to its structural advantage of channel separation, position-sensitive attention, and channel reassembly. C2PSA, short for Cross Stage Partial with Pyramid Squeeze Attention, serves as a core feature enhancement block in the YOLOv11 architecture (Khanam and Hussain, 2024). In tea pest and disease segmentation, this module helps capture early-stage lesion characteristics such as local shape deformation and initial color variations. However, a more detailed analysis reveals two inherent limitations. The position-sensitive attention in PSA models spatial relationships within a restricted receptive field, making it insufficient for lesions that spread across multiple leaf regions or exhibit propagation patterns along physiological textures. Moreover, the subsequent feed-forward network (FFN) only performs linear channel transformation without a multi-scale response mechanism, resulting in boundary artifacts and loss of fine texture details when confronted with lesions of multiple scales and complex vein structures.

**Figure 5:**
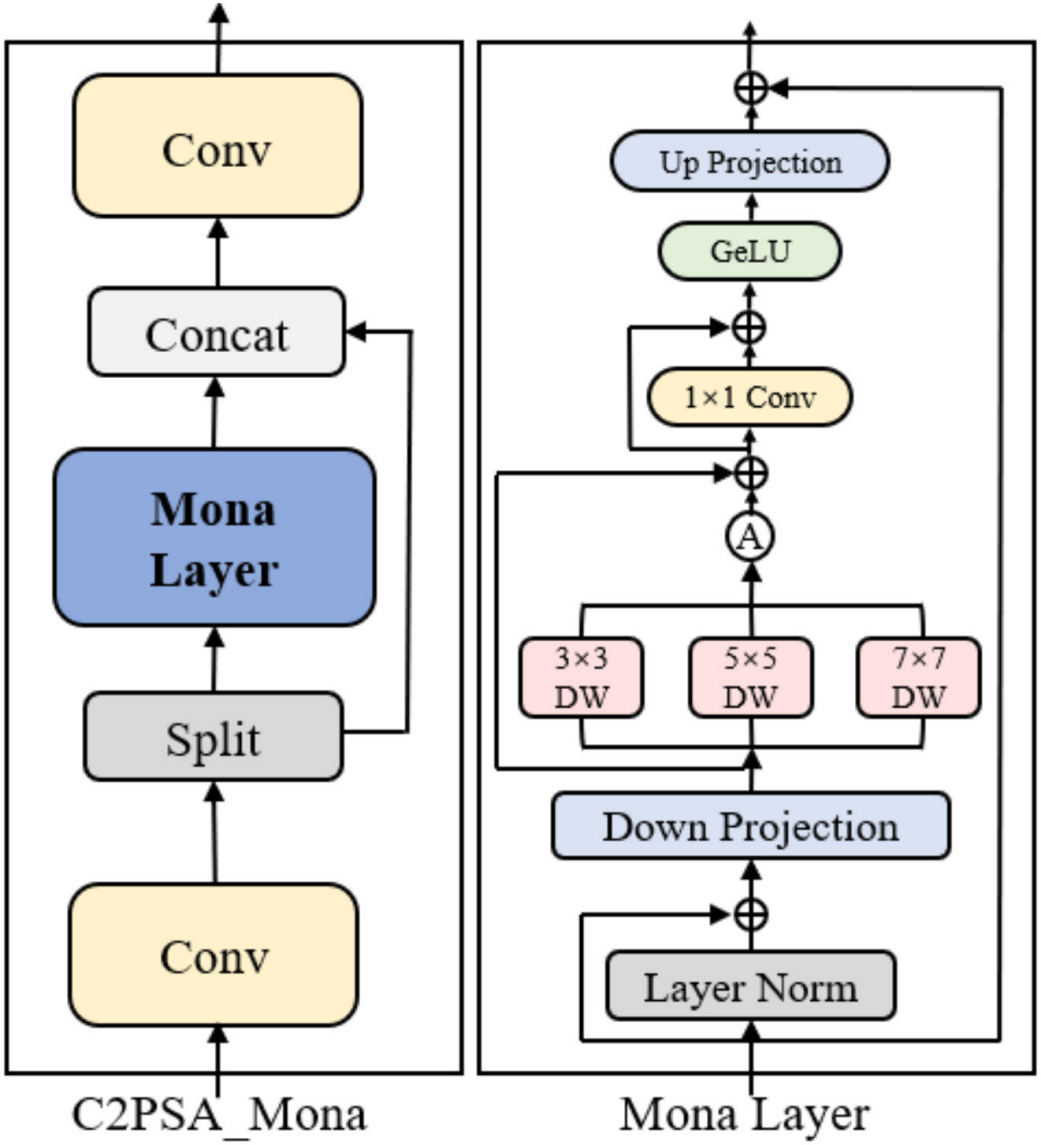
Diagram of the C2PSA_Mona module.

To overcome these constraints, we introduce the C2PSA_Mona module, which integrates the Mona multi-scale depth convolution unit (Yin et al., 2024) into the original C2PSA design. The proposed module follows a three-stage enhancement process, where attention outputs are adapted to multi-scale spatial encoding and then reinforced through nonlinear residual modulation. This considerably strengthens cross-scale representation and improves segmentation stability under intricate lesion textures. Concretely, the Mona sub-module is inserted at two critical junctures: after PSA, where it augments the joint encoding of global structural cues and local lesion patterns, and after the FFN, where it recalibrates multi-scale responses to refine texture boundaries, lesion contour continuity, and small-scale morphological variations. These enhancements allow features to maintain coherence between global structural dependencies and local fine-grained expressions. The architecture of the C2PSA_Mona module is illustrated in **Figure 5**.

The implementation details are as follows. Given an input feature tensor X, the module first follows the channel-split strategy of the original design and applies a 1×1 convolution to perform linear dimensionality reduction. The downsampled feature is computed as shown in Equation 4:

(4) $$X_{down}\;=\;Conv_{1\times 1}^{r}(X),\;\;r\;=\;8$$

where the reduction ratio   r controls channel compression and serves as the basis for subsequent cross-scale operations.

To mitigate the statistical shift between pretrained features and tea-disease imagery, we introduce two learnable scaling factors $\;s_{1}$ and $s_{2}$. that adaptively balance the calibrated response between LayerNorm output and the reduced feature representation. The distribution-adjusted output is formulated as shown in Equation 5:

(5) $$\hat{X}\;=\;s_{1}\cdot \;LayerNorm(X_{down})\;+\;s_{2}\cdot X_{down}$$

which stabilizes the numerical range of the features and enhances robustness against illumination variability and heterogeneous imaging conditions common in leaf acquisition.

After diribution calibration, Mona applies three depthwise convolutions equipped with different receptive fields to perform multi-scale texture encoding. The output is expressed as shown in Equation 6:

(6) $$F_{m}\;=X_{down}\;+\;\frac{1}{3}\;\sum_{k}DWConv_{k\times k}(\hat{X})⊕BN(\cdot ),\;k\in \{3,5,7\}$$

where the residual addition ⊕ maintains steady gradient propagation across the multi-scale branches. The encoded feature $F_{m}$ is then projected into a compact 64-dimensional representation through a 1×1 bottleneck convolution, during which GELU activation is applied to enhance nonlinear expressiveness. This design introduces multi-receptive fusion without expanding channel width, enabling the model to adaptively balance boundary preservation and regional smoothness, thereby improving structural consistency in complex lesion segmentation.

## 3 Results and analysis

### 3.1 Experimental setup

The proposed TDS-YOLO model was trained and evaluated on a workstation running Ubuntu 22.04, using the PyTorch 2.2 deep learning framework with Python 3.8. The hardware platform included an Intel Xeon E5–2699 v4 processor (2.2 GHz), 128 GB RAM, and an NVIDIA RTX 4090 GPU equipped with 24 GB of memory. CUDA 12.1 and cuDNN 8.2 were configured to accelerate parallel computation and neural network training.

All experiments were conducted with an input resolution of 640 × 640 and a batch size of 128. The network was trained for 200 epochs using the SGD optimizer, with the following hyperparameters: an initial learning rate of 0.01, a momentum factor of 0.937, and a weight decay of 5 × 10^−4^. To improve convergence stability, a warm-up phase of approximately three epochs was applied before switching to a cosine annealing strategy for learning rate scheduling. In addition, automatic mixed precision (AMP) training was employed to reduce GPU memory usage while maintaining numerical accuracy.

### 3.2 Model evaluation

To comprehensively assess the effectiveness of the proposed modules within the YOLOv11-seg framework, a series of ablation and comparative experiments were conducted under identical datasets and training configurations. Four commonly used metrics—Parameters, Precision, Recall, and mean Average Precision (mAP)—were selected as the primary evaluation indicators. Precision and Recall are defined in Equations 7 and 8, respectively:

(7) $$P\;=\;\frac{TP}{TP+FP}$$

(8) $$R\;=\;\frac{TP}{TP+FN}$$

where TP (True Positive) denotes correctly detected instances, FP (False Positive) refers to incorrectly detected instances, and FN (False Negative) represents missed detections. Precision reflects the proportion of correct predictions among all detections, whereas Recall measures the proportion of correctly detected targets among all targets that should be detected.

Based on these two metrics, Average Precision (AP) is computed as the area under the Precision–Recall curve. The mean Average Precision (mAP) across all classes is given in Equation 9:

(9) $$mAP\;=\;\frac{\sum AP}{N(Class)}$$

where N(Class)  represents the total number of categories.

In addition, two widely used IoU (Intersection over Union)–based metrics are adopted in our evaluation. mAP@0.5 measures the mean AP at an IoU threshold of 0.50, focusing on whether detected disease regions are correctly localized. mAP@0.5:0.95 computes the average AP over ten IoU thresholds ranging from 0.50 to 0.95 in increments of 0.05, imposing a more stringent and comprehensive assessment on both localization precision and prediction robustness.

### 3.3 Convergence evaluation

Model convergence is a fundamental criterion for assessing the effectiveness of deep learning training, as it reflects whether the model parameters progressively stabilize toward an optimal solution during iterative optimization. By analyzing the evolution of loss functions alongside the variations of key performance metrics during both training and validation, one can comprehensively evaluate the stability, convergence quality, and optimization efficiency of the model. In this work, the convergence behavior of TDS-YOLO is systematically examined based on 16 continuously monitored indicators during training, including eight loss components and eight performance metrics, providing a complete perspective on the model’s training dynamics.

**Figure 6:**
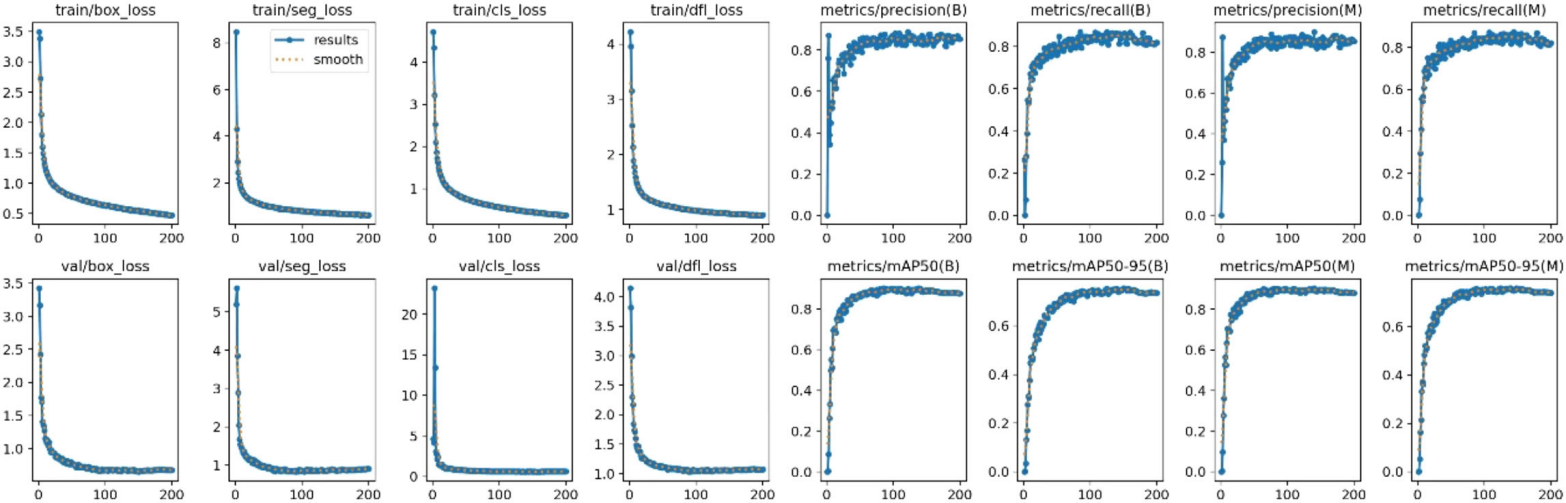
Training indicator curves of the TDS-YOLO model.

As illustrated in **Figure 6**, both the loss functions and performance metrics display clear convergence behavior. During training, the loss values for bounding-box regression, segmentation, classification, and distribution fitting decrease rapidly within the first 100 epochs, showing a reduction of more than 60%, and later stabilize within a low range of approximately 0.5 to 2.0 near the 200th epoch. The validation losses exhibit a similar downward pattern, and the numerical differences between training and validation consistently remain below 0.3, indicating that the model does not suffer from overfitting. In terms of model performance, detection metrics including precision, recall, and mean average precision under IoU 0.50 as well as the stricter IoU range from 0.50 to 0.95 increase rapidly during early training, particularly within the first 100 epochs. For example, mAP50 rises from about 0.20 to 0.85. After this stage, these metrics enter a stable plateau with fluctuations smaller than 0.05, suggesting that the model parameters have converged to an optimal and stable state. Overall, TDS-YOLO achieves simultaneous convergence in both loss reduction and performance improvement within 200 epochs, demonstrating a stable and reliable training process consistent with the expected optimization behavior.

### 3.4 Ablation study

To clarify the individual contributions of each innovative component in the TDS-YOLO model and validate the effectiveness of their designs, ablation experiments were conducted on three core modules: the C3K2_EViM_CGLU feature-enhancement module, the EfficientHead lightweight unified detection–segmentation head, and the C2PSA_Mona multi-scale structural enhancement module. The experiments were performed on the self-constructed dataset, using the number of parameters, precision, recall, and mAP@0.5 as the main evaluation metrics. Four comparison settings were established: (A) a baseline using the original YOLOv11n-seg model, (B) a replacement model in which the C3k2 module was substituted by C3K2_EViM_CGLU, (C) a head-replacement model that retains the original feature hierarchy but replaces the detection head with EfficientHead, and (D) a feature-enhancement model in which the C2PSA module was replaced by C2PSA_Mona. Detailed ablation results are reported in **Tables 2** and **3**.

**Table 2:** Ablation results for object detection.

Model	C3K2_EViM_CGLU	EfficientHead	C2PSA_Mona	Precision	Recall	mAP@.5	Parameter
A				84.1	83.2	88.6	2.84M
B	✓			83.7	84.6	85.5	2.73M
C	✓	✓		**87.2**	81.8	88.4	**2.49M**
D	✓	✓	✓	84.1	**86.3**	**90.1**	2.53M

Bold values indicate the best performance in each column.

**Table 3:** Ablation results for instance segmentation.

Model	C3K2_EViM_CGLU	EfficientHead	C2PSA_Mona	Precision	Recall	mAP@.5	Parameter
A				85.1	84.0	89.3	2.84M
B	✓			85.5	84.7	89.6	2.73M
C	✓	✓		**89.0**	81.7	89.7	**2.49M**
D	✓	✓	✓	84.3	**86.4**	**90.1**	2.53M

Bold values indicate the best performance in each column.

As shown in **Table 2**, the baseline model (A) achieves a precision of 84.1%, a recall of 83.2%, and an mAP@0.5 of 88.6%, with a parameter size of 2.84M. After introducing the C3K2_EViM_CGLU module (B), the Recall improves from 83.2% to 84.6%, indicating that the module effectively strengthens global representation to reduce missed detections. Although the mAP@0.5 experiences a temporary fluctuation (dropping to 85.5%), this step is essential as it reconstructs the feature space to support the subsequent lightweight head. Building further upon this, replacing the original detection head with EfficientHead (C) leads to a significant recovery in performance: precision rises to 87.2% and mAP@0.5 rebounds to 88.4% (comparable to the baseline). Crucially, these results are achieved while drastically reducing the model size from 2.84M to 2.49M. This demonstrates that EfficientHead successfully maintains high-performance multi-scale localization through shared convolutions and structural reparameterization, even with a lighter architecture. When the C2PSA_Mona module is integrated into the full configuration (D), the synergistic effect of the three modules is fully realized. Compared with the baseline, the complete TDS-YOLO achieves a 3.1% improvement in Recall and a 1.5% increase in mAP@0.5, while reducing the parameter count by 0.31M. This confirms that while the individual modules focus on different aspects (Recall enhancement vs. Parameter reduction), their combination leads to an optimal balance. The C2PSA_Mona module provides the final boost through expanded multi-scale receptive fields, enabling superior recognition of lesion morphology and boundary transitions.

Instance segmentation results further validate the effectiveness of the three modules for pixel-level tasks. The baseline model (A) achieves an mAP@0.5 of 89.3%. With C3K2_EViM_CGLU (B), while precision decreases slightly, recall and mAP@0.5 significantly improve, suggesting stronger spatial modeling ability in boundary regions and complex texture zones. When EfficientHead is incorporated (C), precision increases to 90.2% and mAP@0.5 rises to 89.7%, again demonstrating that the lightweight unified head captures multi-scale structural cues more effectively during mask generation. The full integration of C3K2_EViM_CGLU, EfficientHead, and C2PSA_Mona (D) yields the best performance, with improvements of 2.2% in recall and 1.4% in mAP@0.5 for instance segmentation, while maintaining a compact parameter size of only 2.53M.

In conclusion, the proposed TDS-YOLO is specifically tailored for tea leaf disease segmentation and achieves balanced optimization across multiple performance dimensions. The fusion of C3K2_EViM_CGLU, EfficientHead, and C2PSA_Mona results in a synergistic combination of high efficiency, lightweight computation, and high accuracy. The model simultaneously reduces parameter size, preserves real-time inference capability, and enhances both detection and segmentation precision, demonstrating the task adaptability and collaborative benefits of the three innovative modules. Such characteristics meet the dual practical requirements of fine-grained segmentation and real-time performance for tea disease monitoring in real-world agricultural environments.

### 3.5 Comparative experiments

#### 3.5.1 Comparison between TDS-YOLO and YOLOv11 during training

**Figure 7:**
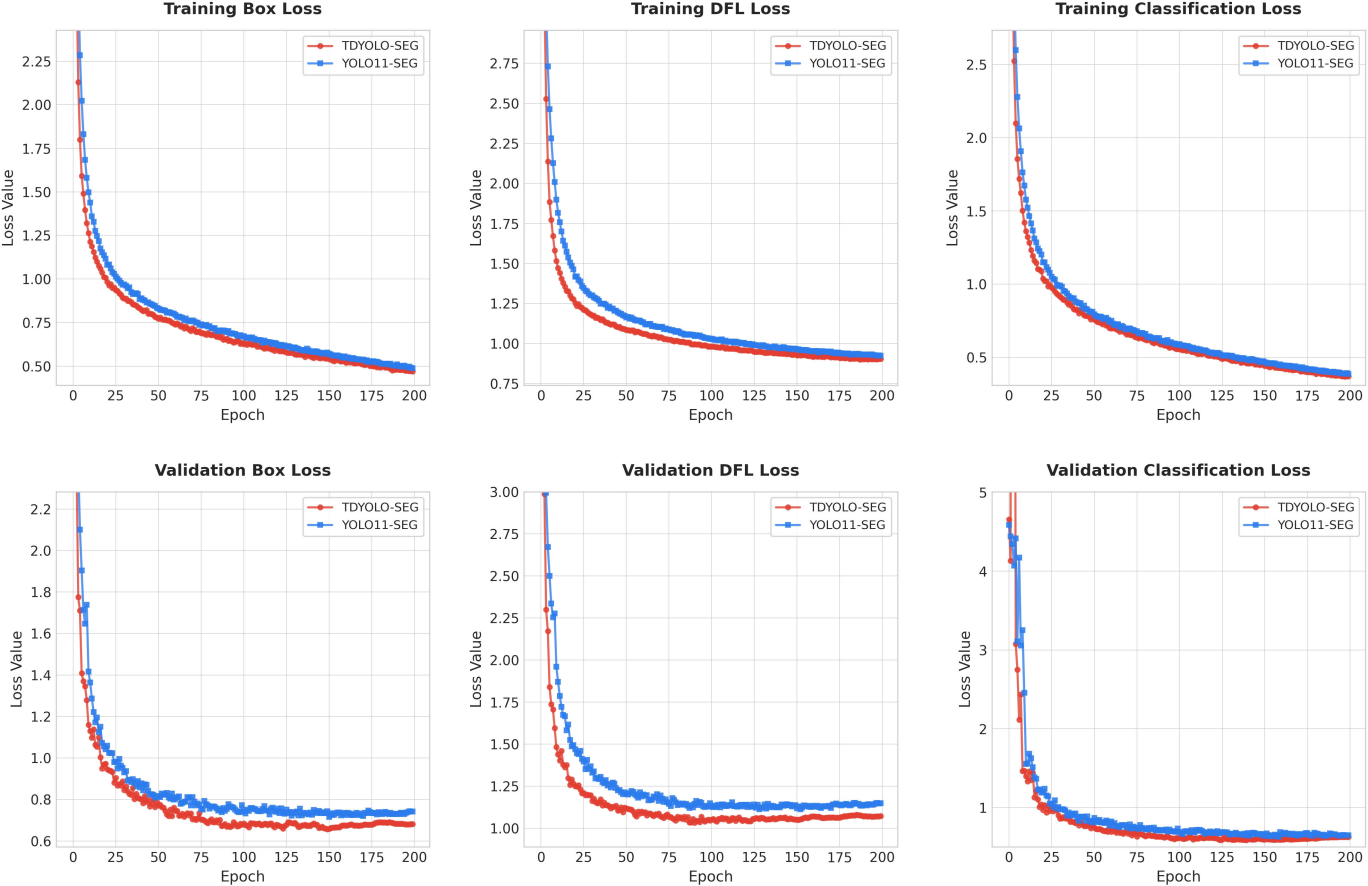
Loss comparison between TDS-YOLO and YOLOv11 during training.

To compare the training behavior of the original YOLOv11 model with that of the proposed TDS-YOLO, a loss-curve analysis was performed. The evolution of the loss values for both models throughout the training process is presented in **Figure 7**.

The loss curves during training clearly show that the improved model exhibits faster and more stable convergence across three core loss components that govern detection quality: box regression loss, distribution fitting loss, and classification loss. The regression loss drops more steeply and reaches a lower stable region earlier than YOLOv11, indicating a stronger ability to learn lesion boundaries and their geometric properties. The distribution fitting loss also decreases more efficiently and converges to a smaller value, demonstrating that the model achieves a more precise estimation of bounding box distribution and learns the localization error pattern more effectively. Likewise, the classification loss becomes lower and smoother, reflecting a higher discriminative capability when distinguishing visually similar tea leaf disease categories.

During validation, the trend remains consistent. The regression loss remains below that of YOLOv11 throughout training and stabilizes at a lower level, suggesting that the proposed model maintains consistent lesion boundary prediction on unseen samples. The distribution fitting loss in validation decreases with smaller fluctuations, which implies stronger generalization of the learned target distribution and reduced sensitivity to variations in leaf texture or illumination. The classification loss is significantly lower and becomes more stable in later epochs, showing that the model is more robust in recognizing fine-grained disease differences and less prone to overfitting.

#### 3.4.2 Comparative performance across different models

To further evaluate the advantages of the proposed TDS-YOLO, we conducted a comparative experiment against several mainstream models, including YOLOv5 (Olorunshola et al., 2023), YOLOv8 (Reis et al., 2023), YOLOv10 (Wang A. et al., 2024), YOLOv11 (Khanam and Hussain, 2024), YOLOv12 (Tian et al., 2025), YOLOv13 (Lei et al., 2025), Faster R-CNN (Ren et al., 2016), Mask R-CNN (He et al., 2017), RT-DETR (Zhao et al., 2023). Since YOLOv5 and YOLOv10 do not support instance segmentation, their results are reported only for detection performance, whereas the remaining models are compared in both detection and segmentation tasks. The experimental outcomes are summarized in **Table 4** for detection performance and **Table 5** for segmentation performance.

**Table 4:** Comparison of detection performance across different models.

Model	Parameter	Precision	Recall	mAP@.5	mAP@.5:.95
Faster R-CNN	59.2M	40.9%	22.0%	19.3%	15.1%
RT-DETR	15.4M	84.7%	85.6%	89.2%	68.2%
YOLOv5	**2.18M**	85.3%	80.8%	88.2%	71.9%
YOLOv8-seg	2.94M	84.1%	83.7%	88.9%	75.0%
YOLOv10	2.7M	83.3%	83.0%	88.4%	72.9%
YOLOv11-seg	2.84M	84.1%	83.2%	88.6%	74.2%
YOLOv12-seg	2.77M	**86.2%**	78.6%	87.0%	73.1%
YOLOv13-seg	2.71M	83.3%	82.3%	87.9%	74.3%
TDS-YOLO	2.53M	84.1%	**86.3%**	**90.1%**	**75.1%**

Bold values indicate the best performance in each column.

**Table 5:** Comparison of segmentation performance across different models.

Model	Parameter	Precision	Recall	mAP@.5	mAP@.5:.95	FPS
Mask R-CNN	44.0M	88.9%	87.6%	89.6%	76.3%	34
YOLOv8-seg	2.94M	85.0%	84.6%	89.1%	74.2%	79
YOLOv11-seg	2.84M	85.1%	84.0%	89.3%	75.2%	88
YOLOv12-seg	2.77M	**87.1%**	79.7%	88.0%	73.6%	91
YOLOv13-seg	2.71M	84.2%	88.3%	88.7%	75.1%	90
TDS-YOLO -seg	**2.53M**	84.3%	**86.4%**	**90.1%**	**75.9%**	**96**

Bold values indicate the best performance in each column.

As shown in **Table 4**, TDS-YOLO achieves the best overall performance in the object detection task. With a parameter size of only 2.53M, it is significantly more lightweight than classical two-stage detectors such as Faster R-CNN (59.2M) and transformer-based RT-DETR (15.4M), as well as YOLOv8-seg, YOLOv10, YOLOv11-seg, YOLOv12-seg, and YOLOv13-seg, demonstrating superior model compactness and suitability for lightweight deployment. In terms of core detection metrics, TDS-YOLO obtains the highest Recall at 86.3% and the highest mAP@0.5 at 90.1%. Compared with the stronger YOLO-based baselines, its Recall increases by 3.1% over YOLOv11-seg, 7.7% over YOLOv12-seg, and 4.0% over YOLOv13-seg; similarly, its mAP@0.5 rises by 1.5%, 3.1%, and 1.2%, respectively. Although Faster R-CNN and RT-DETR represent strong detection baselines, Faster R-CNN suffers from extremely low Recall (22.0%) and mAP@0.5 (19.3%) under this dataset, indicating limited adaptability to small and densely distributed tea disease targets, while RT-DETR achieves competitive accuracy but at the cost of substantially higher parameter complexity. Although YOLOv5 and YOLOv10 produce competitive results at mAP@0.5, their performance degrades more noticeably at the stricter IoU threshold represented by mAP@0.5:0.95. This indicates that their localization accuracy deteriorates more rapidly under harsher evaluation conditions. In contrast, TDS-YOLO maintains a higher mAP@0.5:0.95 of 75.1%, highlighting its superior robustness in precise localization, small-object detection, and complex background scenarios.

According to **Table 5**, TDS-YOLO-seg achieves the strongest overall performance in the instance segmentation task. It reaches a Recall of 86.4 percent, an mAP@0.5 of 90.1 percent, and an mAP@0.5:0.95 of 75.9 percent, reflecting improved lesion coverage, more accurate boundary delineation, and enhanced segmentation stability under complex field conditions. These improvements are particularly relevant for fine-grained disease regions with irregular morphology and weak texture contrast. Among one-stage YOLO-based segmentation models, TDS-YOLO-seg consistently provides higher segmentation accuracy while maintaining a compact model size of 2.53 million parameters. In comparison, the classical two-stage instance segmentation framework Mask R-CNN employs 44.0 million parameters and achieves an mAP@0.5 of 89.6 percent, indicating that similar segmentation accuracy can be obtained with substantially reduced model complexity in the proposed approach. In terms of inference efficiency, TDS-YOLO-seg achieves an average processing speed of 96 frames per second, which exceeds the speeds of YOLOv8-seg, YOLOv11-seg, YOLOv12-seg, and YOLOv13-seg, whose inference rates range from 79 to 91 frames per second. By contrast, Mask R-CNN processes approximately 34 frames per second under the same experimental settings. These results demonstrate that TDS-YOLO-seg effectively balances segmentation accuracy and computational efficiency, making it suitable for real-time tea disease monitoring in resource-constrained deployment scenarios.

In the category-level segmentation comparison shown in **Table 6**, TDS-YOLO-seg demonstrates a clear performance advantage for the Tea-bud-blight class. Specifically, the YOLOv11-seg baseline achieves an mAP@0.5 of 80.0, whereas TDS-YOLO-seg reaches 83.4, yielding a 3.4 percentage point improvement. Such a margin reflects a meaningful and quantifiable distinction in instance segmentation performance.

**Table 6:** Comparison of mean segmentation accuracy across different tea diseases.

Classification	YOLO11-seg mAP@0.5	TDS-YOLO -seg mAP@0.5
tea_leaf_blight	89.9	89.6
Tea-bud-blight	80	83.4
Tea_ring_rot	92.9	**93.3**
Tea_algae_leaf_spot	94.4	94

Bold values indicate the best performance in each column.

From the perspective of task characteristics and model adaptability, Tea-bud-blight lesions are typically small in size, sparsely distributed, and often exhibit blurred boundaries with healthy tissues. These properties impose higher demands on a model’s ability to perceive small targets and capture fine-grained visual cues. The enhanced feature fusion mechanisms in TDS-YOLO-seg allow the model to more effectively align with the morphological and textural attributes of Tea-bud-blight lesions. As a result, TDS-YOLO-seg surpasses YOLOv11-seg on this specific disease type, further validating its adaptive superiority in fine-grained, disease-specific segmentation scenarios.

### 3.6 Analysis of experimental results

Classification performance serves as a key indicator for evaluating the practical usefulness of disease recognition models, as it reflects how accurately and robustly different disease types can be identified. A comprehensive evaluation can be obtained by examining several analytical curves and indicators, including the F1–confidence relationship, the Precision–Recall profile, and the mean Average Precision. In this section, we analyze the classification stability and overall performance of the proposed model based on its recognition results across different categories of tea leaf diseases.

**Figure 8:**
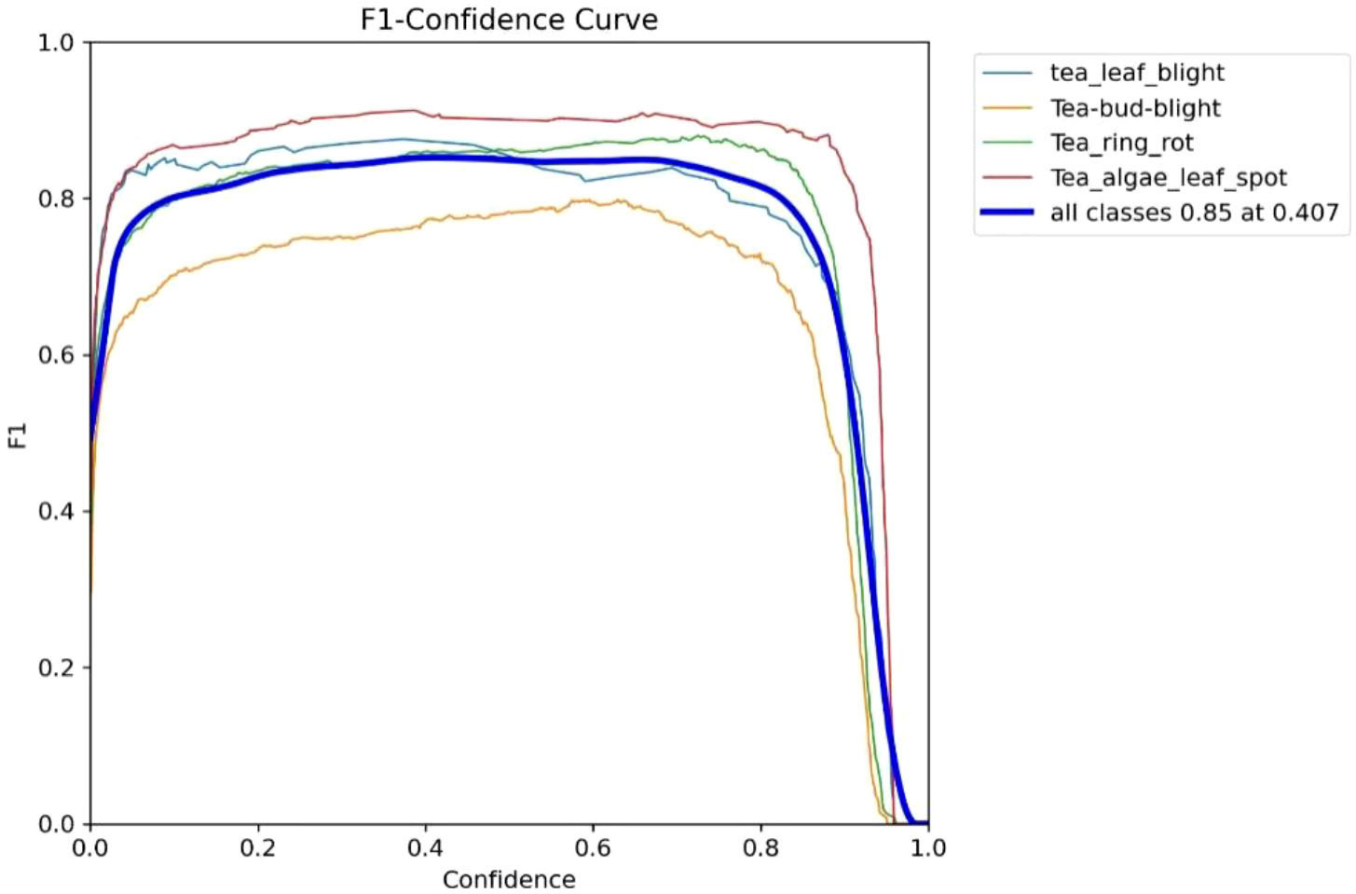
F1–confidence curve.

As shown in **Figure 8**, the F1 curves for all disease categories remain at a high level (F1 ≥ 0.8) within the confidence interval of 0.0 to 0.8, and only drop sharply when the confidence approaches 1.0. This indicates that the model maintains stable classification performance across a wide confidence threshold range. The peak value of the overall F1 curve (0.85) occurs at a confidence threshold of 0.407, which represents the optimal balance point at which the model achieves the best classification performance across all tea disease categories.

**Figure 9:**
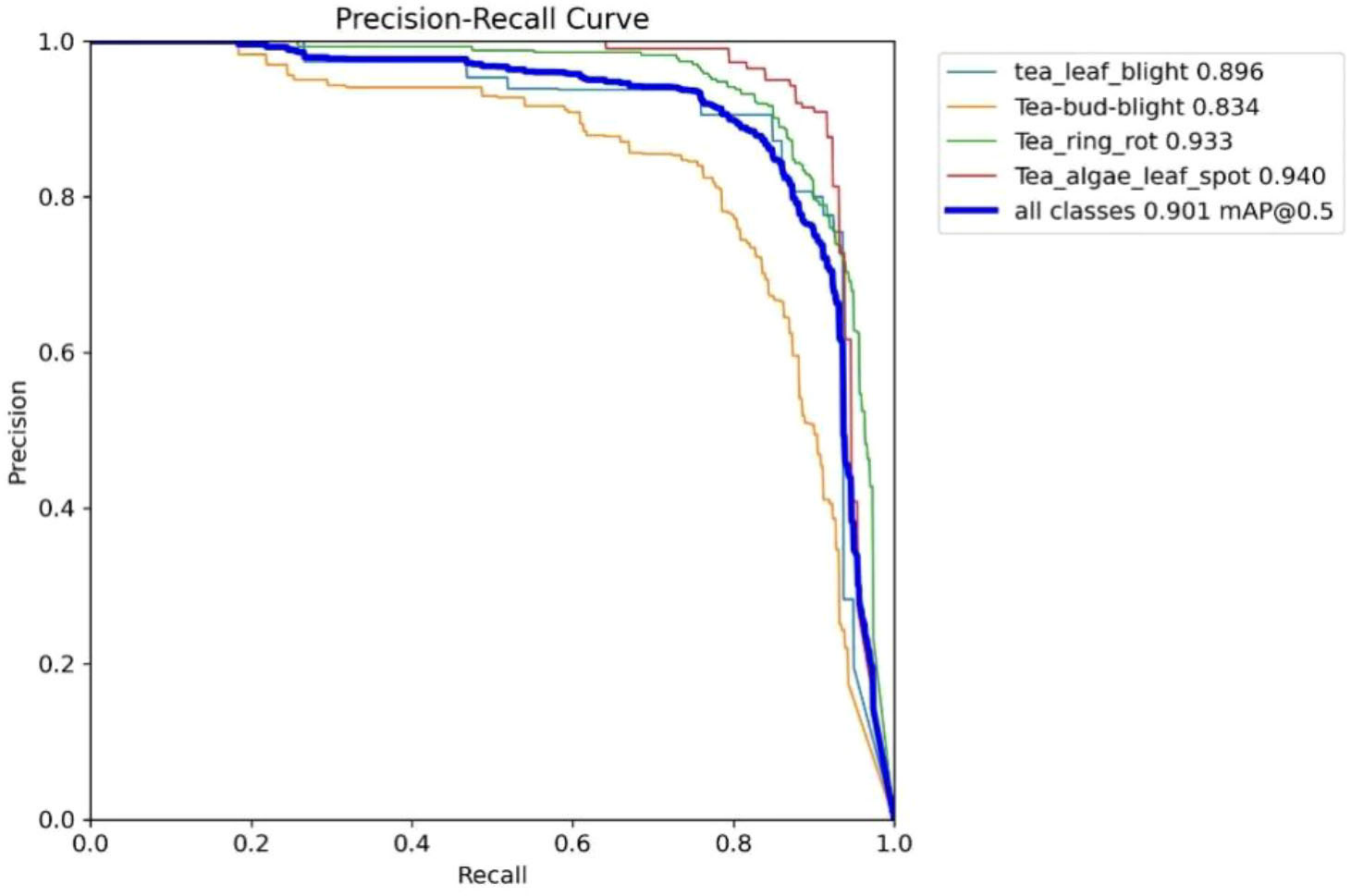
Precision–Recall curves for different disease categories.

As illustrated in **Figure 9**, the Precision–Recall curves of all disease classes are concentrated in the high-precision and high-recall region. Among them, Tea_algae_leaf_spot (mAP@0.5 = 0.940) and Tea_ring_rot (mAP@0.5 = 0.933) achieve the best classification performance, while Tea-bud-blight (mAP@0.5 = 0.834) is slightly lower but still remains at a comparatively high level. The overall mAP@0.5 reaches 0.901, further confirming that the model offers strong comprehensive classification capability for tea leaf diseases, with a well-balanced trade-off between precision and recall across all categories.

**Figure 10:**
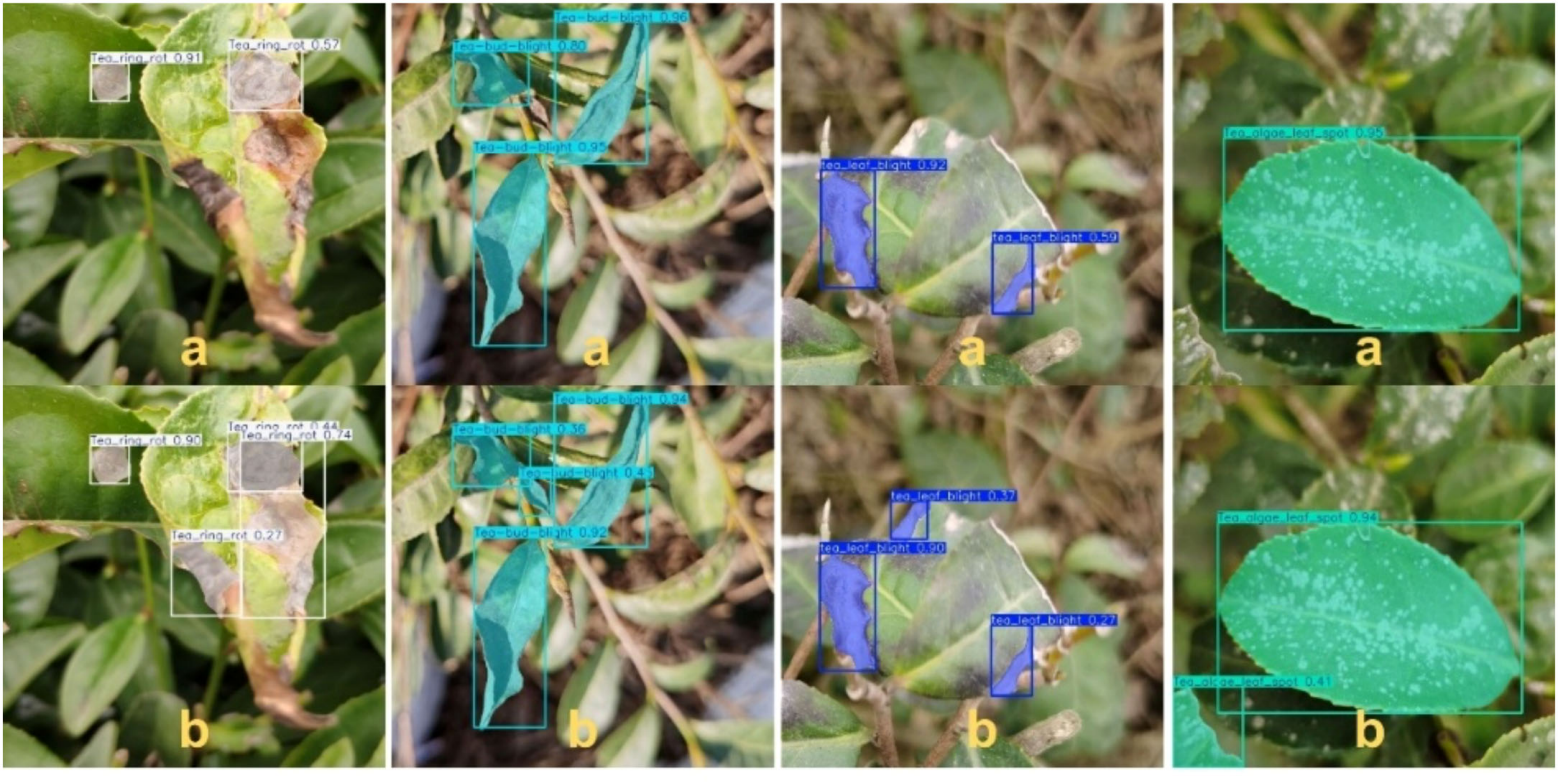
Comparison of real-scene detection results: **(a)** YOLOv11 (baseline), **(b)** TDS-YOLO (proposed).

In this experiment, both the original YOLOv11 model and the proposed TDS-YOLO model were tested in a real application scenario. The detection outcomes are illustrated in **Figure 10**, where (a) shows the results produced by YOLOv11 and (b) presents the detection results obtained using TDS-YOLO.

As shown in the visual results of **Figure 10**, the original YOLOv11 model is capable of performing basic detection of the four major tea diseases, including tea leaf blight, tea bud blight, tea ring rot and tea algal leaf spot. However, its performance decreases noticeably when dealing with complex backgrounds or fine-grained lesion structures. Specifically, YOLOv11 often produces incomplete boundary localization or shifted bounding boxes in cases of leaf blight and bud blight, especially when lesion color gradually transitions into surrounding tissues or when the edge presents slight corrosion-like patterns, resulting in unstable localization. For diseases such as tea ring rot and tea algal leaf spot, which exhibit circular textures or light-colored patches, YOLOv11 tends to miss small lesions or confuse them with specular reflections on leaf surfaces.

**Figure 11:**
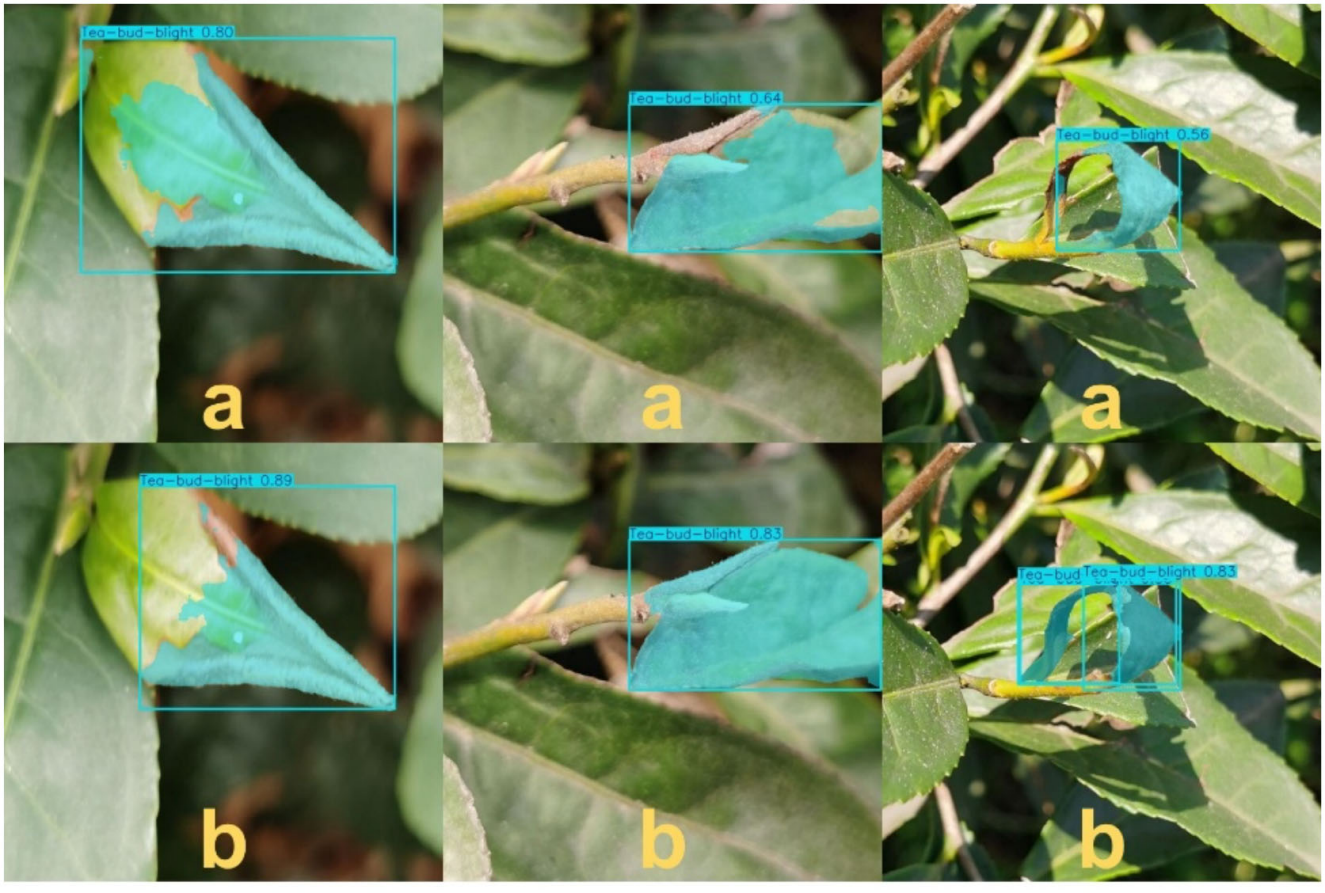
Boundary-level comparison of tea bud blight segmentation results. **(a)** YOLOv11 (baseline). **(b)** TDS-YOLO (proposed).

**Figure 11** provides a focused boundary-level comparison of segmentation results for tea bud blight under complex field conditions. As shown in (a), the baseline model tends to produce incomplete or imprecise masks in regions with irregular lesion shapes or strong background interference, resulting in under-segmentation or boundary deviation, particularly near leaf veins and branch junctions. In contrast, the proposed TDS-YOLO in (b) generates more continuous and compact segmentation masks with improved boundary alignment. The refined results demonstrate better coverage of slender and weakly textured lesion regions, indicating enhanced boundary-level segmentation stability beyond the overall visual comparisons shown in **Figure 10**.

In contrast, the proposed TDS-YOLO demonstrates significantly improved boundary fitting and more robust lesion perception across all four disease categories. Benefiting from the global–local joint modeling ability of the C3K2_EViM_CGLU module, the efficient decoding capability of EfficientHead, and the multi-scale texture enhancement achieved by C2PSA_Mona, TDS-YOLO more accurately captures irregular lesion contours, intralesional texture variations, and subtle chromatic gradients. As a result, the predicted bounding boxes align more closely with the true lesion regions. The improved model effectively suppresses background interference and enhances the distinction between diseased and healthy tissues, thereby reducing both missed detections and false positives.

## 4 Conclusions

With the growing demand for intelligent monitoring of tea plantation diseases and pests, the development of a model that simultaneously achieves real-time inference, lightweight deployment, and high-precision segmentation holds substantial importance for smart tea-garden management and agricultural quality control. In this work, we propose TDS-YOLO, a segmentation-oriented architecture tailored for tea leaf disease detection. By introducing systematic enhancements across backbone feature extraction, cross-scale semantic learning, and instance-level mask decoding, the model addresses several key limitations observed in traditional YOLO-based frameworks under complex natural conditions, including insufficient global representation, imprecise lesion boundary modeling, and unstable recognition of small disease spots. Through the integration of the C3K2_EViM_CGLU feature extractor, the lightweight EfficientHead decoder, and the multi-scale enhancement block C2PSA_Mona, TDS-YOLO effectively distinguishes fine-grained lesion textures from background noise and significantly improves detection and segmentation accuracy while maintaining a compact architecture.

Experimental results demonstrate that TDS-YOLO, with only 2.53M parameters, achieves the highest Recall and mAP@0.5 in object detection, and delivers the best mAP@0.5 and mAP@0.5:0.95 performance for instance segmentation, outperforming recent models such as YOLOv8-seg, YOLOv11-seg, YOLOv12-seg, and YOLOv13-seg. These results confirm that the collaboration between the three proposed modules substantially strengthens global–local contextual modeling, fine-scale structural representation, and high-resolution boundary estimation. Furthermore, visual detection outcomes show that TDS-YOLO accurately delineates lesion edges, identifies subtle chlorosis and small corrosion marks, and avoids misclassifications caused by glare or complex backgrounds, thereby reducing false alarms and missed detections. This demonstrates strong applicability and deployment potential in real-world tea plantation environments. Overall, TDS-YOLO achieves a synergistic optimization of efficiency, lightweight computation, and high segmentation precision, providing a practical and scalable solution for intelligent tea disease monitoring.

Looking forward, the methodology proposed in this work is not limited to tea production and can be extended to disease detection and precise segmentation in other crops, such as tomatoes, cucumbers, and grapes. By further integrating unmanned aerial vehicles, mobile platforms, and agricultural Internet-of-Things systems, future research could enable a more automated and real-time monitoring pipeline, supporting the development of digital agriculture and the construction of smart tea-growing ecosystems.

## Data Availability

The original contributions presented in the study are included in the article/supplementary material. Further inquiries can be directed to the corresponding author/s.
